# Reversing cancer stemness

**DOI:** 10.18632/aging.100846

**Published:** 2015-11-15

**Authors:** James Block, Fanyan Meng, Guojun Wu

**Affiliations:** Karmanos Cancer Institute, Department of Oncology, Wayne State University School of Medicine, Detroit, MI 48202, USA

**Keywords:** cancer stem cell, PDGFR, cancer metastasis, EMT, breast cancer

Metastasis is the major factor responsible for the lethality of malignant breast cancer in human patients. Although various targeted and non-targeted therapies can occasionally control the progress of breast cancer, a significant portion of patients develop resistance to chemotherapy and experience metastatic recurrence.

The epithelial to mesenchymal transition (EMT), a key developmental program in embryogenesis, has been found to be closely intertwined with the occurrence of metastasis in various human cancers. EMT can be prompted by the expression of multiple transcriptional factors and is controlled by several signaling pathways including TGF-β, Wnt and Notch signaling. Moreover, a link between EMT and the onset of epithelial stem cell-like properties has been suggested. However, the mechanism underlining this relationship remains unrevealed, and whether cancer stemness is a consequence of EMT or they are two parallel phenomena refecting cell plasticity remains unknown.

Towards the goal of understanding breast cancer metastasis, our group performed a cross-species expression profiling and identified Foxq1 as an EMT- and metastasis-promoting gene in breast cancer [1]. Following this discovery, Foxq1 expression has been shown to promote EMT and metastasis in a wide array of human cancers [2-4]. In line with the previously-mentioned link between EMT and stemness, we demonstrated that ectopic expression of FOXQ1 led to an increase in the stem-like phenotype and CD44+/CD24− population consists of over 90% of cells. This increase in the stem cell population correlated with the induction of EMT. Mechanistically, we identified the receptor tyrosine kinases PDGFR α and β as downstream targets of FoxQ1 [5].

Our study showed that knockdown of PDGFR α and β significantly decreased cell proliferation, migration and invasion in HMLE/FoxQ1 cells. The effects were greatest when both α and β were knocked down. Knockdown of PDGFR α and β in HMLER/FoxQ1 cells decreased lung metastases in vivo. Moreover, Knockdown of α and β, or β alone, decreased the CD44+/CD24− phenotype by 25% without reversing the FoxQ1-induced EMT at the molecular and morphological levels. These results strongly suggest that FoxQ1's role as a promoter of the CSC's phenotype is regulated in part by PDGFR activity.

We used the RTK inhibitor imantinib to examine the effects of pharmacologic silencing of PDGFR expression. Although imantinib targets multiple RTK's, we demonstrated that only PDGFR α and β expression were significantly altered in FoxQ1-upregulated cells. High doses of imantinib significantly inhibited cell proliferation, while the low-dose treatment markedly inhibited cell migration and invasion in vitro. Either pharmacological treatment or genetic manipulation of PDGFR α and β expression significantly increased the sensitivity of HMLE/FoxQ1 cells to doxorubicin or paclitaxel treatment. In vivo, doxorubicin with imantinib treatment inhibited tumor growth by approximately 80%. However, the use of either drug alone did not significantly inhibit xenograft growth, demonstrating that the two drugs exhibited a synergistic effect.

Our study demonstrates that EMT and stemness properties are not controlled by identical gene programs, at least in some cell lines. Inhibiting PDGFR α and β significantly reduced the stemness properties of HMLE/FoxQ1 cells without impacting the mesenchymal-like phenotype of those cells. Since this inhibition correlated with a marked decrease in malignancy, our study suggests that the acquisition of stem-like properties may drive malignancy to a greater degree than EMT alone. Our gene expression profiling indicates that Foxq1 regulates a wide spectrum of downstream gene targets, significantly repressing cell adhesion and cell junction genes and promoting ECM genes (unpublished data). Taken together, our data suggests that EMT and stemness are two closely related cell characteristics induced by related or identical transcription factors, but ultimately controlled by different downstream gene programs.

In line with our results, a recent study showed that low levels of Twist1 are essential for tumor initiation, maintenance, and stemness independent of its EMT-inducing activity [6]. In another study, two cancer stem cell populations were found to co-exist in human cancer cells and demonstrate distinct epithelial and mesenchymal properties [7]. It is currently unclear if cancer stem cells are capable of transitioning between these two populations. The conditions to trigger this switch and whether this switch is consistent with EMT and its role in cancer progression and metastasis remain to be explored.

Our study also indicates that cancer stemness can be partially reversed by targeting PDGFRs. The high levels of imantinib employed in our study would not be applicable in the clinic in terms of its low specificity and toxicity. Consequently, a specific PDGFR α and β inhibitor with lower toxicity than imantinib would increase the clinical potential of our discovery. Further studies must be done to identify other pharmacological targets that synergize with the stemness-promoting activity of PDGFRs. Reversing cancer stemness, together with conventional chemotherapy, could provide an ideal approach for prevent cancer recurrence and metastasis by eradicating both the bulk tumor cells and the cancer stem cells with self renewal capability.

## References

[1] Zhang H (2011). Cancer research.

[2] Abba M (2013). Molecular cancer research: MCR.

[3] Feng J (2014). Oncotarget.

[4] Xia L (2014). Hepatology.

[5] Meng F (2015). Cancer research.

[6] Beck B (2015). Cell stem cell.

[7] Liu S (2014). Stem cell reports.

